# SMCT1 has a low affinity to PDZ domain containing 1 protein

**DOI:** 10.17912/micropub.biology.001502

**Published:** 2025-02-19

**Authors:** Qingyang Zhang, Jacob Clinton, Kristina Westerlund, Carsten Mim

**Affiliations:** 1 Department of Protein Science, KTH Royal Institute of Technology, Stockholm, Stockholm, Sweden; 2 Department of Biomedical Engineering and Health Systems, KTH Royal Institute of Technology, Stockholm, Stockholm, Sweden

## Abstract

Sodium-coupled monocarboxylate transporter 1 (SMCT1) is a membrane transporter abundantly expressed in colon, kidney, thyroid, brain; and silenced in cancer cells. It transports monocarboxylic acids with little specificity into cells. Based on pulldown experiments, it was proposed that the scaffolding protein PDZ Domain Containing 1 (PDZK1) regulates its surface expression and increases SMCT1’s transportation efficiency. Here, we performed pull-down assays, Surface Plasmon Resonance (SPR), and Micro Scale Thermophoresis (MST) to evaluate the affinity between SMCT1 and two PDZ domains in PDZK1. Our results show that SMCT1 binds to these PDZ domains. However, the estimated equilibrium dissociation constants are higher than in canonical PDZ domains and likely physiological not relevant.

**Figure 1. SMCT1 interaction with PDZ1 and PDZ3:**
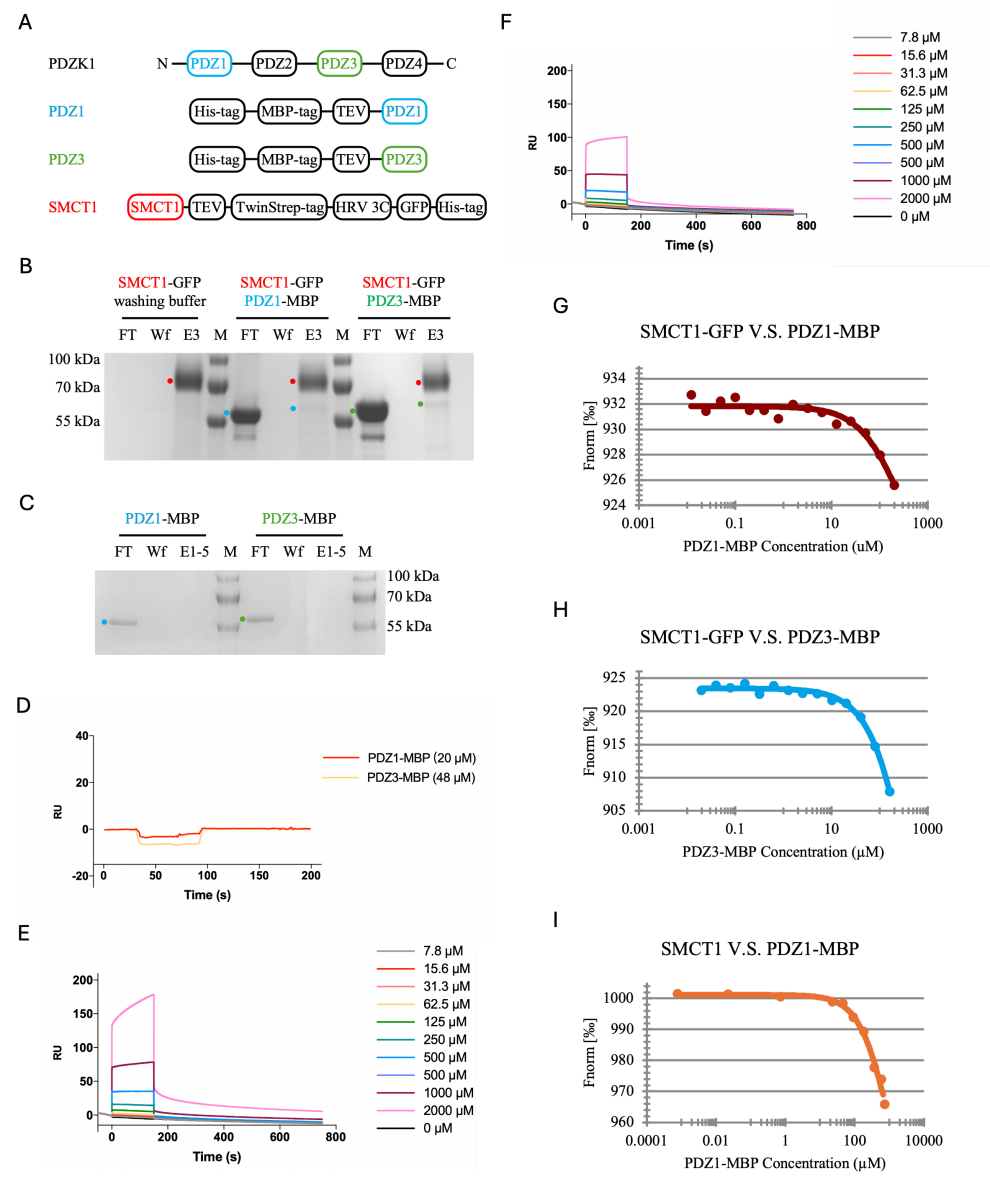
**(A)** design for constructs used in this study. **(B)** SDS-PAGE of the PDZ1-MBP and PDZ3-MBP pull-down assay by immobilized SMCT1-GFP on TwinStrepXT resin. Unbound PDZ1-MBP or PDZ3-MBP was shown in the flowthrough (FT) fractions. The final wash (Wf) removed unspecific bound PDZ1-MBP or PDZ3-MBP. In (E3) was the third eluate fraction (E3) with the strongest GFP signal. ( **C)** Control for unspecific PDZ1-MBP and PDZ3-MBP binding to TwinStrepXT resin, with the same conditions as in B. **(D)** SPR sensorgram where PDZ1-MBP (red line) and PDZ3-MBP (yellow line) was perfused over a chip immobolized with PEPTIDE. **(E)** SPR sensorgram of serial-diluted PEPTIDE (0-2000 μM) perfused over immobilized PDZ1-MBP. **(F)** SPR sensorgram of serial-diluted PEPTIDE (0-2000 μM) perfused over immobilized PDZ3-MPB. **(G)** MST data and fit for the sample containing SMCT1-GFP (0.8 μM) and PDZ1-MBP (12 nM-0.2 mM). **(H)** MST data and fit for the sample containing SMCT1-GFP (0.8 μM) and PDZ3-MBP (19.5 nM-0.16 mM). **(I)** MST data and fit for the sample containing SMCT1 (2.3 μM) and PDZ1-MBP (0.75 nM-0.7255 mM).

## Description

Monocarboxylic acids such as lactate, pyruvate, ketone bodies and vitamin B3 are important metabolites. Sodium-coupled monocarboxylate transporter 1 (SMCT1) transports one monocarboxylate together with two sodium ions across the membrane. SMCT1 is abundantly expressed in colon, kidney, thyroid and brain. In colon, it absorbs short chain fatty acids; in kidney it reabsorbs lactate and nicotinate; in thyroid, it releases iodide; in brain it transports lactate and ketone bodies into neurons as energy source (Ganapathy et al., 2008; Jha & Morrison, 2020) . Pulldown assays identified a PDZ motif (T-R-L) at the SMCT1 C-terminus to mediate interaction with the scaffolding protein PDZ Domain Containing 1 protein (PDZK1) (Otsuka et al., 2019; Srivastava et al., 2019) . PDZK1 may not only in increase SMCT1’s surface expression but also SMCT1’s transport activity for nicotinate (Srivastava et al., 2019) . PDZK1 contains four PDZ domains (PDZ1, PDZ2, PDZ3 and PDZ4, illustrated in figure 1A), but only PDZ1 and PDZ3 interacts to SMCT1. Given that pulldown and yeast two-hydride assays are problematic for judging interactions (Mackay et al., 2007) , we aim to quantify the binding affinity between SMCT1 with PDZ1 and PDZ3 by performing Surface Plasmon Resonance (SPR) and Micro Scale Thermophoresis (MST). While we confirmed that SMCT1 can bind to both PDZ1 and PDZ3, SPR and MST showed that the affinity is much higher than those described for common PDZ domain interactions.

First, we performed a pull-down assay to confirm the documented binding between SMCT1-GFP with PDZ1-MBP and PDZ3-MBP. Binding assay was carried out by purifing SMCT1 with TwinstrepXT beads, which we chose to minimize unspecific binding. We used these beads to pull down PDZ1-MBP and PDZ3-MBP in solution. Wash and elution fractions were analyzed by SDS-PAGE. We detected that a small fraction of PDZ1-MBP, and PDZ3-MBP respectively, co-eluted with SMCT1-GFP ( Fig. 1B ). To estimate unspecific binding we applied PDZ1-MBP and PDZ3-MBP to untreated TwinstrepXT beads ( Fig. 1C ).

The pulldown experiments implied a low affinity, which we wanted to quantify by SPR, which has been routinely used to measure affinity in PDZ domains. Therefore, we synthesized a peptide (11 amino acid, W-Q-S-G-K-S-N-G-T-R-L, denoted as PEPTIDE hereinafter) containing the PDZ motif from SMCT1. Generally, the affinity between the PDZ domain and the PDZ motif is in the 10 μM range (Gianni et al., 2005; Lee & Zheng, 2010) . We perfused PDZ1-MBP (20 μM) and PDZ3-MBP (48 μM) over immobilized PEPTIDE, but we could not detected binding to PEPTIDE for either PDZ1-MBP or PDZ3-MBP ( Fig. 1D ). Our inability to measure any affinity, might be due to the obstruction of PEPTIDE’s PDZ motif by the immobilization through amino moieties, either/or at the N-terminus and the single lysine. Consequently, we immobilized PDZ1-MBP, and PDZ3-MBP respectively, on the chip. The sensorgram showed that the PEPTIDE bound to both PDZ1-MBP ( Fig. 1E ) and PDZ3-MBP ( Fig. 1F ). The association rate and dissociation rate were fast, which is consistent with binding features of other PDZ-motif peptides with PDZ domains (Flores et al., 2008) . A steady-state affinity fitting analysis was not successful, likely because the equilibrium dissociation constant (K _D_ ) is beyond the tested concentration range. Both SPR experiments imply a low affinity.

The SPR experiments prompted us to think that any possible interaction may involve more than the short sequence at the end of SMCT1. So, we used MST to measure the affinity between full-length SMCT1 with a GFP-tag and PDZ1 and PDZ3 MBP constructs. The binding curves for SMCT1-GFP/PDZ1-MBP and SMCT1-GFP/PDZ3-MBP indicate some binding, observable by a drop-off in fluorescence ( Fig. 1G, H ). But we were not able to reach saturation within tested concentration range. As such, we can only estimate a lower limit for K _D_ of about 265.14 μM (SD= 0.56%) and 639.07 μM (SD=0.49%) for SMCT1-GFP/PDZ1-MBP and SMCT1-GFP/PDZ3-MBP interaction, respectively. In order to investigate the influence of GFP tag on the binding, we tested fluorescently labelled SMCT1 without GFP tag, yet with the same results as SMCT1-GFP ( Fig. 1I ).

In summary, we employed three methods to investigate the interaction between SMCT1 and PDZ1/3. Although, our pulldown with purified SMCT1 shows binding to PDZ1 and PDZ3, we demonstrated that the affinity is between one and two orders of magnitude higher than typical PDZ-based affinities. It could be that our assays are sensitive to detergents or other factors that we have not controlled. However, our pulldown results can be explained that ligand density and bead geometry may generate locally high concentrations of a ligand for the target to bind (Hintersteiner et al., 2012; Wissmueller et al., 2011) . We suggest here that the PDZK1-SMCT1 interaction is likely physiologically irrelevant, except in special cases, where PDZK1 is locally enriched at membrane.

## Methods

Peptide sythnesis

Peptide (W-Q-S-G-K-S-N-G-T-R-L) of last 10 amino acid from C-terminus of SMCT1 and one additional W was synthesised (Westerlund et al., 2024) and purified by RP-HPLC (Agilent, California, USA). The identity and purity of the peptide was confirmed by 4800 MALDI TOF/TOF™ Analyzer (Applied Biosystems, Massachusetts, USA).

Protein expression and purification

SMCT1-GFP was expressed in sf9 cells. The Bac-to-Bac ^TM^ Baculovirus Expression System user guide (Gibco ^TM^ ) was referred to perform the expression. In brief, full-length human SMCT1 (UniprotID: Q8N695) was cloned into pFastBac1 vector (Life Technologies, California, United States) with Tobacco etch virus (TEV) protease recognition sequence (E-N-L-Y-F-Q-S), TwinStrep-tag, human rhinovirus 3C (HRV 3C) protease recognition sequence (L-E-V-L-F-Q-G-P) and green fluorescent protein (GFP)-tag at the C-terminus and transformed into DH10 Multibac E. coli (Geneva Biotech, Geneva, Switzerland) to make the recombinant Bacmid construct. Sf9 cells (Life Technologies, California, USA) were routinely maintained in our laboratory. Sf9 cells were grown in suspension at 28°C at 110 rpm in sf-900 ^TM^ III medium (Life Technologies, California, United States) in Corning ^®^ Erlenmeyer shake flasks (non-baffled with vent cap). Sf9 cells (Life Technologies, California, United States) are transfected by Bacmid construct using X-tremeGENE™ 9 DNA Transfection Reagent (Roche Diagnostics GmbH, Mannheim, Germany) to produce virus. Sf9 cells at a density of 2 × 10^6 cells/mL were infected with optimal virus amount determined by test expression. Cells were harvested at viability around 80% and re-suspended with lysis buffer 1 (150 mM NaCl, 50 mM Tris at pH 7.5, 5% Glycerol and cOmplete™ protease inhibitor). Cells were then lysed by sonication with 65% power for 8 min of 5 s on and 25 s off. Cell debris were removed by centrifugation at 4000 g for 20 min at 4°C. Membranes from the supernatant were harvested by ultracentrifugation (Ti50.2 rotor) at 32000 rpm for 1 h. Membrane pellet were homogenized with Dounce tissue grinder and solubilized in lysis buffer with 2% DDM and 0.4% CHS for 2 h at room temperature. Unsolubilized material was removed with ultracentrifugation (Ti50.2 rotor) at 32000 rpm for 1 h. The supernatant was adjusted to 1 mM EDTA and bound to 2 mL (bed volume) Strep-Tactin ^®^ XT (IBA Lifesciences, Göttingen, Germany) using a batch protocol for 2 h at 4°C. The resin was washed with 20x bed volume of washing buffer (150 mM NaCl, 100 mM Tris at pH=8.0, 0.1% DDM, 0.02% CHS and 1 mM EDTA). 150ul of beads were kept for Pull-down assay. Protein was eluted with washing buffer containing 50 mM biotin. The elution fractions were pooled and concentrated with 50kDa cutoff. The concentrated protein was further purified by size-exclusion chromatography (SEC) using a Superose 6 Increase 10/300 GL column (Cytiva, Uppsala, Sweden) with washing buffer without EDTA. Peak fractions were pooled and concentrated. SDS-PAGE were performed to examine the purity.

PDZ1 and PDZ3 were cloned into pETM-41 vector (a gift from the EMBL protein production facility) with Maltose-Binding Protein (MBP)-tag and TEV protease recognition sequence at N-terminus and transformed into TUNER cell for expression. When OD reached 0.7, cell was induced with 0.5 mM IPTG for 3 hours at 37°C. Cell was harvested and re-suspended in lysis buffer 2 (200 mM NaCl, 20 mM Tris at pH 7.4, 1 mM EDTA) with cOmplete™ protease inhibitor. Cell was then lysed by sonication with 65% power for 8 min of 5 s on and 25 s off. Cell debris was removed by centrifugation and the supernatant was bound to 1ml MBP-Trap-HP column (Cytiva, Uppsala, Sweden). The resin was washed with 20x bed volume of lysis buffer. Protein was eluted with lysis buffer with 10mM maltose. The protein was concentrated and further purified by size-exclusion chromatography (SEC) using a Superose 6 Increase 10/300 GL column using lysis buffer without EDTA. Peak fractions were pooled and concentrated. SDS-PAGE were performed to comfirm the purifity.

Fluorescent labelling

SMCT1 was incubated with TCEP for 2 hours and followed by adding the Alex Fluor ^TM^ 488 C _5_ Maleimide (Invitrogen, Oregon, USA) into the mixture and incubalted at 4°C overnight. Glutathione was added to consume excess thiol-reactive reagent. The remaining TCEP, dye and glutathione was removed by PD10 desalting colume (Cytiva, Uppsala, Sweden). Fluorescent labelled protein was concentrated for later use.

Pull-down assay

For each pull-down assay, 50 uL TwinStrepXT beads binding with SMCT1 is used and placed in a hand packed gravity column. After washing, beads were incubated with 550ul washing buffer, 550 uL PDZ1 (0.9 mg/ml) and 550 uL PDZ3 (0.96 mg/ml), respectively, overnight. Flowthrough was collcted. The beads were washed with 20x bed volume of washing buffer. Final wash was collected. Protein was eluted with washing buffer added with 50 mM biotin. Flowthrough, final wash and elution fraction were loaded on SDS-PAGE gel. Negative control was performed by loading the empty TwinStrepXT beads with PDZ1 and PDZ3 separately overnight.

SPR

Biacore T200 (GE Healthcare, Uppsala, Sweden) was used for biosensor analysis. The PEPTIDE were immobilized on surfaces of flow cell (FC) 2 on a CM5-chip by amine coupling in sodium acetate buffer at pH 5.5. The PDZ1 and PDZ3 were immbolized in FC2 and FC3 on another CM5-chip by amine coupling in sodium acetate buffer at pH 4.0. The final immobilization levels of peptide are 146.4 RU (FC2), PDZ1 are 7187.1 RU (FC2) and PDZ3 are 4267.6 RU (FC3). Reference flow cell was created on FC1 by activation and deactivation of a surface. PBS with 0.1% Tween-20 was used as running buffer and for dilution of the analytes. All experiments were performed at 25°C with a flow-rate of 30 μl/min. the contact time was 150 s and dissociation time was 600 s. The chips were regenerated by injection of 10 mM HCl for 30 seconds. The binding kinetics were analyzed by Biacore evaluation software.

MST

SMCT1 and PDZ domain proteins were centrifuged to remove aggregates before pre-mixed into eppendorf tubes. The mixture was aspirated into glass capillaries and measured under Monolith NT.115 instrument (NanoTemper Technologies, Munich, Germany). The experiment was performed with blue LED color for both GFP-tagged and fluorescent labelled SMCT1. The concentration of SMCT1-GFP used in this experiment was 0.8 μM, and the concentration of fluorescent labelled SMCT1 used in this experiment was 2.3 μM. A serial dilution was performed for both PDZ domain proteins with MBP tag (from 0.2 mM to 12 nM for PDZ1-MBP, and 0.16 mM to 19.5 nM for PDZ3-MBP) and PDZ1 (from 0.7255 mM to 0.75 nM). Data analysis was done in NT Analysis Software using Thermophoresis mode.

## Reagents

**Table:** 

**Plasmid**	**Genotype**	**Source**
pFastBac1		Life Technologies
pETM-41		Gift from the EMBL Protein Expression and Purification Core Facility
		
Strain		Source
Tuner (DE3)		Novagen
DH 10 MultiBac		Geneva Biotech
Sf9		Life Technologies
		
**Other reagents**	**Usage**	**Source**
PreScission protease	Protease to remove tag	Laboratory-produced
TEV protease	Protease to remove tag	Laboratory-produced
Strep-Tactin ^®^ XT	Purify SMCT1	IBA Lifesciences
MBP-Trap-HP column	Purify PDZ1 and PFZ3	Cytiva
Alex 480	Fluorescent dye for SMCT1 labelling	Invitrogen
sf-900 ^TM^ III medium		Life Technologies
X-tremeGENE ^TM^ 9 DNA Transfection Reagent		Life Technologies
